# Predictive Value of CT-Based Radiomics in Distinguishing Renal Angiomyolipomas with Minimal Fat from Other Renal Tumors

**DOI:** 10.1155/2022/9108129

**Published:** 2022-05-28

**Authors:** Zhiwei Han, Yuanqiang Zhu, Jingji Xu, Didi Wen, Yuwei Xia, Minwen Zheng, Tao Yan, Mengqi Wei

**Affiliations:** ^1^Department of Radiology, Xijing Hospital, Air Force Medical University, Xi'an, Shaanxi, China; ^2^Huiying Medical Technology Co., Ltd., Room A206, B2, Dongsheng Science and Technology Park, Haidian District, Beijing 100192, China; ^3^Department of Radiology, Xi'an XD Group Hospital, Xi'an, Shaanxi, China

## Abstract

**Objectives:**

This study is aimed at determining whether CT-based radiomics models can help differentiate renal angiomyolipomas with minimal fat (AMLmf) from other solid renal tumors.

**Methods:**

This retrospective study included 58 patients with a postoperative pathologically confirmed AMLmf (observation group) and 140 patients with other common renal tumors (control group). Non-contrast-enhanced CT and contrast-enhanced CT data were evaluated. Radiomics features were extracted from manually delineated volume of interest (VOIs). The least absolute shrinkage and selection operator (LASSO) regression was used for feature screening. Five classifiers, including logistic regression, multilayer perceptron (MLP), support vector machine (SVM), *k*-nearest neighbor (KNN), and logistic regression (LR), were used, with leave-out validation (128 training, 60 testing). The diagnostic performance of the classifier was evaluated and compared by receiver operating characteristic curve (ROC) analysis.

**Results:**

Among the 1029 extracted features, prediction models of AMLmf were composed, by 2, 10, 4, and 9 selected features for precontrast phase (PCP), corticomedullary phase (CMP), nephrographic phase (NP), and excretory phase (EP), respectively. Models of CMP and NP achieved adequate performance after using MLP classifier, with prediction accuracy of 0.767 (AUC 0.85, sensitivity 0.76, and specificity 0.78) and 0.783 (AUC 0.83, sensitivity 0.79, and specificity 0.78), respectively. MLP model of features selected from the combination of the all features had the best diagnostic performance (accuracy 0.8500, sensitivity 0.8095, specificity 0.9444, and AUC 0.9193).

**Conclusions:**

Radiomics features may help to distinguish benign AMLmf from common malignant kidney masses, which may contribute to the selection of interventions for renal tumors.

## 1. Introduction

Most renal tumors are asymptomatic and are found incidentally (1). Among them, renal cell carcinomas (RCCs) are the most common, and 85% of renal masses smaller than 4 cm are reported to be RCCs (2, 3). Most malignant renal masses require active intervention of surgical resection or radiofrequency ablation. Angiomyolipoma (AML) is the most common benign solid renal neoplasm which can be diagnosed accurately by identifying the intratumoral fat component. However, for AML with minimal fat (AMLmf), the diagnosis is challenging as it does not contain any fat cells nor contain an insufficient amount of fat cells to provide a computed tomography (CT) image-based diagnosis (4–6). Therefore, AMLmf are often misdiagnosed as RCC and these patients often have surgeries, which would not only increase their economic and psychological burden but also increase the workload of hospitals and waste the cost of medical insurance (7, 8).

Using morphological and enhancement features, Sung found the predictive values were around 80%, which is not very satisfactory (9). Using logistic regression analysis, Zhang et al. found several parameters are valuable in differentiating AMLmf from RCCs, such as unenhanced attenuation characteristic and intratumoral vessels (10). A comparable study using multidetector computed tomography (MDCT) found the long-to-short axis ratio, attenuation and enhancement degree precontrast phase (PCP), corticomedullary phase (CMP), nephrographic phase (NP), and excretory phase (EP) showed significant differences between AMLmf from RCCs (11). Although many studies and efforts have been made on the differential diagnosis of AMLmf and other malignant masses (12), it is still difficult in practice.

In recent years, artificial intelligence and radiomics analysis techniques have provided new methods for image analysis. By extracting and analyzing the radiomics features of renal lesions on routine CT images, it is possible to distinguish between benign and malignant tumors of the kidney (13–16). However, the accuracy and specificity of texture analysis are inconsistent in various reports, and the sample size is too small to get convincing outcomes. In the current study, we included large sample size patients of AML with minimal fat (AMLmf) and other various pathological types of renal tumors, who had been operated in our hospital in the past ten years. The CT images of tumors were retrospectively analyzed and modeled using radiomics techniques for differential diagnosis. We hypothesized that radiomics strategy combined with large dataset will guarantee fine differentiation of the two renal tumors.

## 2. Materials and Methods

The study was approved by the ethics committee of the hospital. Because this was a retrospective study based only on medical records and all data were analyzed in our hospital, patient consent was waived.

### 2.1. Patient Selection

The patients were selected from the pathological and radiological databases of our hospital from January 2010 to October 2018. We searched for any CT investigations for renal masses performed before surgery or biopsy. The CT examinations included four phases of precontrast phase (PCP), corticomedullary phase (CMP), nephrographic phase (NP), and excretory phase (EP) and were performed within two weeks before surgery. Pathological examination was available for each patient and included both histology (hematoxylin-eosin-safran staining) and immunohistochemistry. Patients with renal lesions containing radiologically visible fat or inadequate CT image quality were excluded.

**Figure 1:**
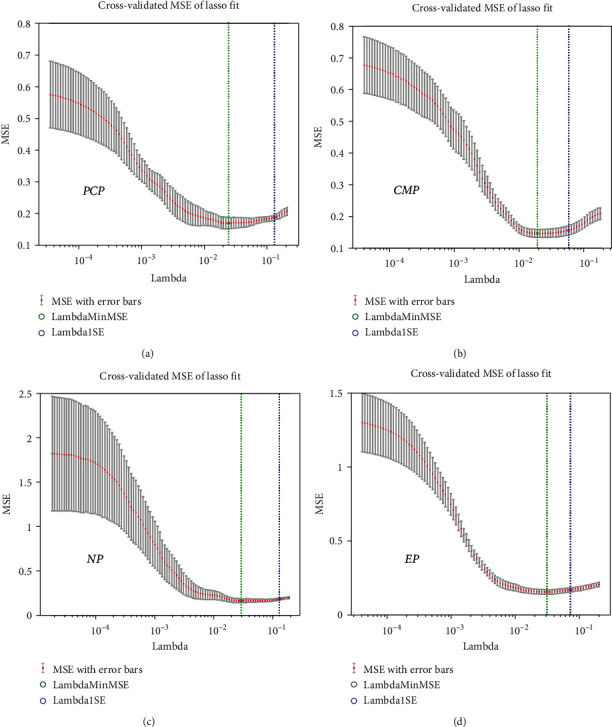
Least absolute shrinkage and selection operator (LASSO) was used for feature screening and dimension reduction. PCP: precontrast phase; CMP: corticomedullary phase; NP: nephrographic phase; EP: excretory phase.

Between January 2010 and October 2018, 318 patients underwent surgical removal of renal masses with preoperative CT scanning and with minimal fat on CT images were included (Figure 1). We excluded patients with inadequate image quality and incomplete scanning phases (*n* = 120). Fifty-eight patients with AMLs with minimal fat and 140 patients with other renal tumors were finally included.

### 2.2. CT Technique

All patients underwent CT scans on multislice spiral CT devices (SOMATOM, TOSHIBA, GE, UNITED IMAGING) using a 4-stage protocol: precontrast phase and then the CMP (30 to 40 s after beginning the iodine contrast injection), NP (70 to 120 s), and excretory phase (3 to 6 min). The contrast medium was injected intravenously into the brachial vein (100 ml, not exceeding 2 ml/kg patient body weight; injection rate: 2.5 to 3.5 ml/s).

### 2.3. Segmentation of the Volume of Interests from Images

The DICOM images of the 198 patients were transferred into the radiomics platform (Big Data Intelligent Analysis Cloud Platform, Huiying Medical Technology Co., Ltd., Beijing, https://mics.radcloud.cn/).

**Figure 2:**
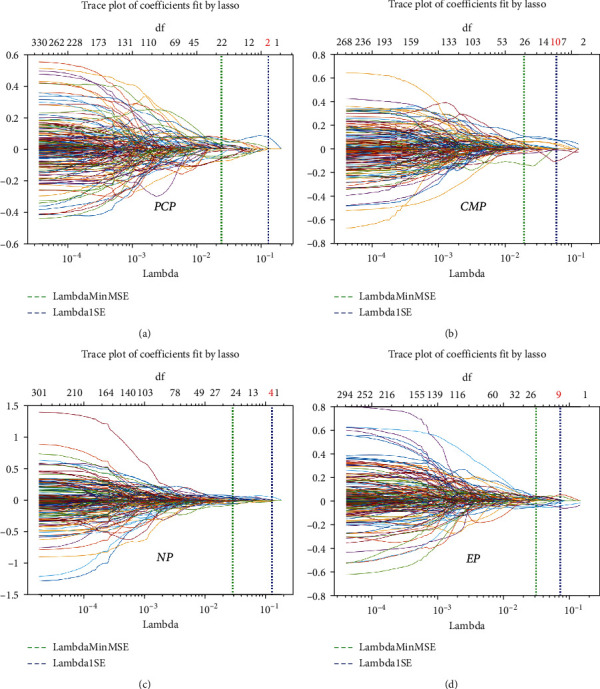
The number of features selected for each phase: 2, 10, 4, and 9 features were selected, for PCP, CMP, NP, and EP, respectively. PCP: precontrast phase; CMP: corticomedullary phase; NP: nephrographic phase; EP: excretory phase.

One reader within eight years of experience in urological imaging delineated the lesion boundaries at all slices of each phase as volumes of interest (VOIs) based on the difference in attenuation and enhancement pattern between the lesion and normal renal parenchyma. Lesions on the PCP usually have lower or equal attenuation and have various patterns of enhancement, compared with renal parenchyma. The outer margin of an exophytic lesion could be easily distinguished from the surrounding fat. If the lesion contour in the PCP was unclear, the PCP phases were referred. Another reader reviewed the VOIs, and if he disagreed with the first reader, a senior radiologist was consulted for the final decision. First, the images of 20 patients were randomly selected for training contour drawing, and then, the interested areas of all patients were drawn (Figure 2). To minimize the CT intensity variations, before feature extraction for VOIs, we normalized by standard deviation [*μ* − 3*σ*, *μ* + 3*σ*], and the platform then automatically analyzed all delineated VOIs and put out data of 1029 features.

### 2.4. Feature Extraction and Screening

First-order statistics, shape-based features, and texture features deriving from gray-level cooccurrence matrix (GLCM), gray-level run-length matrix (GLRLM), gray-level size zone matrix (GLSZM), and neighborhood gray-tone difference matrix (NGTDM) were extracted for each VOI. Also, we applied Laplacian, logarithmic, exponential, and wavelet filters to images and then extracted features based on the filtered image. A total of 1029 radiomics features were extracted. The features were consistent with the imaging biomarker standardization initiative (IBSI) (17). In this study, the least absolute shrinkage and selection operator (LASSO) was used for feature screening and dimension reduction. In high-dimensional data, the LASSO regression method has been proved to be effective and efficient (18, 19). The model coefficients were compressed by selecting the optimal harmonic parameter *λ* in the model by 10-fold cross-validation, and the coefficients of the unrelated variables were reduced to zero while retaining the variables of nonzero coefficients. Finally, several or a dozen significant highly correlated features were retained for further analysis.

### 2.5. Machine Learning for Model Building

As the value range of feature may vary greatly, for example, the value of feature 1 is 1000~2000, and the value of feature 2 is 0.1~0.2, which may affect the contribution of feature to the model. To ensure the convergence of the training model, the value range of features should be within the same scale, so we standardized the value of features (range, 0-1). The selected features were constructed into a prediction model, using leave-out validation (128 training and 60 testing). Five models including logistic regression, multilayer perceptron (MLP), support vector machine (SVM), *k*-nearest neighbor (KNN), and logistic regression (LR) were used to select the classifier with the best diagnostic performance. Model predictive capability is assessed using the receiver operating characteristic curve (ROC) analysis and area under the curve (AUC) as well as accuracy, sensitivity, and specificity.

## 3. Results

### 3.1. Patient Characteristics

The study included 198 patients (111 males and 87 females) aged 22~84 years (mean age, 54.84 ± 12.46 years), of which 58 were AMLs with minimal fat and 140 were other common types of renal tumors, including clear cell RCCs (*n* = 63), chromophobe RCCs (*n* = 32), papillary RCCs (*n* = 31), and oncocytomas (*n* = 14).

### 3.2. Selected Radiomics Features and Machine Learning Models

A total of 1029 radiological features, including 19 first-order statistics, 15 shape-based features, 59 texture features, and features extracted from filtered data, were extracted from each VOI. The texture features included features of GLRLM (*n* = 16), GLCM (*n* = 27), and GLSZM (*n* = 16).

**Figure 3:**
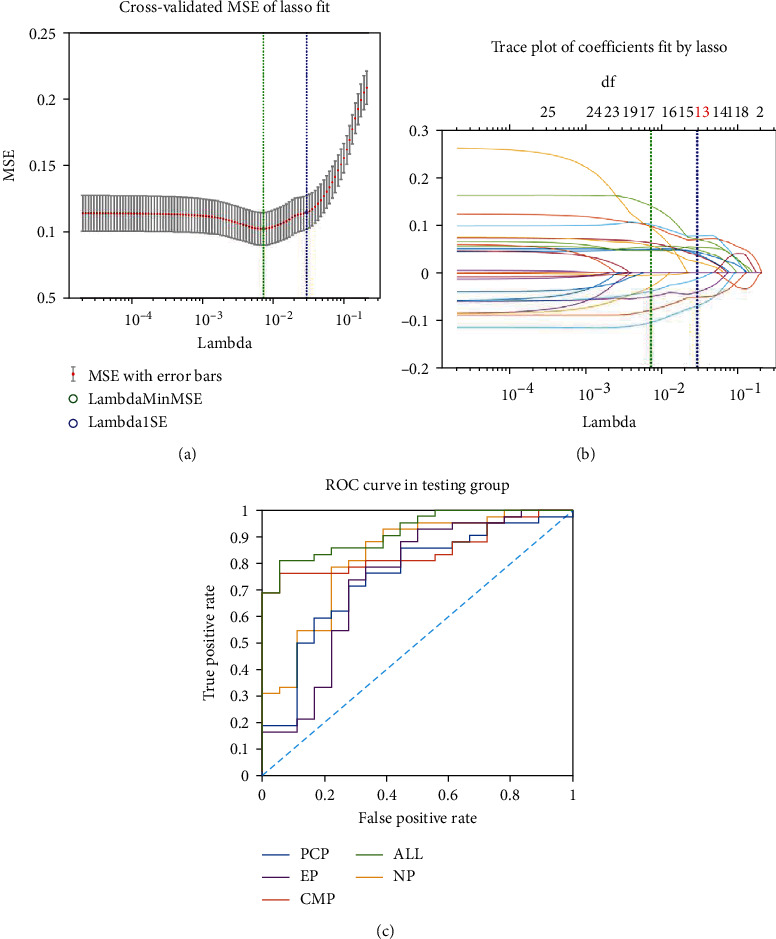
(a) Least absolute shrinkage and selection operator for the combination of the four phases. (b) Thirteen features were selected from the combination of the all features. (c) ROC for each phase and the combination of the four phases using MLP model. MLP: multilayer perceptron.

The LASSO regression was used to reduce the data dimension. From the extracted features of PCP, CMP, NP, and EP, 2, 10, 4, and 9 features were selected, respectively (Figures 1 and 2). Thirteen features were selected from the combination of the all features (Figures 3(a) and 3(b)).

The classification performance of the models was explored by ROC. The optimal model was MLP, as shown in Figure 3(c). MLP model of features selected from the combination of the all features had the best diagnostic performance (accuracy 0.8500, sensitivity 0.8095, specificity 0.9444, and AUC 0.9193) (Table 1).

## 4. Discussion

The detection rate of renal masses has increased in the last decades owing to the widespread use of abdominal imaging. Although RCC accounts for the majority of renal masses (20, 21), 20% of all surgically treated cases are benign, and half of these are AMLs with minimal fat, resulting in unnecessary invasive treatment (22–24). AML is pathologically composed of fat, smooth muscle, and vascular components in different proportions (25). AMLs with minimal fat are mainly composed of smooth muscle and vascular components, and there is no visible fat.

At present, CT is the primary method for diagnosing renal masses, and CT diagnosis of renal angiomyolipoma mainly depends on the intramass visible fat. However, this is not available in cases of AMLs with minimal fat. Current imaging methods have not yet been sufficient to identify AMLs with minimal fat. Radiomics may provide new diagnostic pathways by obtaining and analyzing a large number of features from images. Recent studies have shown that radiomics is important in identifying tumor heterogeneity in several kinds of tumors. Because AMLs with minimal fat is relatively rare compared to renal clear cell and renal papillary cell carcinoma, radiomics studies of renal tumors are focused on relatively common renal tumors. Studies on the most frequently occurring are in (2, 3, 5, 9).

A total of 1029 features for each CT imaging phase were extracted from each manually delineated VOI, and 2 to 10 valid features were selected using the LASSO method. We also combined the extracted features of all phase and ultimately selected thirteen optimal features using the LASSO method. We found that in the five data groups of MSCT phases (PCP, three postcontrast phases, and combined), the ultimately selected features contained first-order statistics and GLCM features, suggesting variations in the gray scale distribution of each entity. First-order statistics describe the distribution of voxel intensities of preset VOI regions through commonly used and basic metrics. The statistics used in this study were mean (mean of image intensity values), median (median of image intensity values), skewness (asymmetry of intensity distribution), and 10 percentile. By calculating the correlation of gray scale between two points of a certain distance and a certain direction in the image, GLCM reflects the comprehensive information of the image in direction, interval, and change. Maximum probability was included in the effective GLCM features of all the five groups, indicating that the distribution of gray value between adjacent pixels of different types of lesions was different.

Among the five classifiers of logistic regression, KNN, LR, MLP, and SVW, the classifier with the best performance was MLP, whose AUC were above 0.73 to 0.85 in the dataset of the four phases. The AUC of combined all phases reached 0.9193, with an accuracy of 0.8500. At present, there were many studies on the use of machine learning to assist in the identification of renal tumors (14, 26, 27), and there were also many studies focused on the differential diagnosis of AMLs with minimal fat (13, 28, 29). Among them, the accuracy of the SVM classifier for identifying AMLs with minimal fat and clear cell carcinoma was 72.3% and 72.1%, respectively. Our study had similar results. In our study, multiphase data were analyzed, and the regions of interest were obtained by volumetric delineation, which was more reflective of the spatial characteristics of tumors than some single-layer delineation, making the extracted features more valuable for differential diagnosis. In the process of VOI delineation, two abdominal radiologists with ten years of experience were present at the same time. When there was inconsistency, a senior abdominal radiologist was consulted. We applied five classifiers, adopted leave-out cross-validation (128 training and 60 testing), for the feature selection of multiphase and combined phase data, making the selected features more valuable for differential diagnosis.

Our study was retrospective and had a large time span; there was some variance in CT scanner, scanning protocol, and contrast agents. The data was not completely unified. Although our results were not the best, they were more consistent with the actual clinical situation, and the results were more suitable for popularization. Among the patients who underwent surgery for renal masses within eight years, a total of 75 cases of AMLs with minimal fat with preoperative misjudgment were found. All these patients underwent unnecessary surgical treatment, which was caused by the limitations of diagnosis. Only 58 cases were included in the study, and the remaining cases were excluded from the study due to incomplete data. The disease accounts for a lower proportion of patients undergoing surgery for renal masses, which may lead to misclassification. Some studies adopted data amplification method to solve this problem (28, 30, 31). In the absence of data amplification, we found little difference between the predicted results and the actual results.

Unlike some literatures (13, 20, 28, 32), we did not separately compare AMLs with minimal fat with a distinct pathological type of renal tumors. We compared AMLs with minimal fat with common renal tumors as a whole, which was consistent with the real diagnostic situation. When we found a small renal mass with minimal fat, clear cell carcinoma was the most likely diagnosis. However, it was difficult to determine the diagnosis; when the mass was located superficially, the attenuation of the unenhanced scan was relatively high, the enhanced scan had no typical enhancement pattern of wash-in and washout, and there were no surrounding fat stranding sign, increased vessels, or lymph nodes. Although relatively rare, we need to consider the possibility of benign lesions, especially AMLmf, with the highest proportion of benign lesions, and thus, the identification of AMLmf with renal carcinoma is consistent with the diagnostic procedure. Finally, we included 198 patients with renal tumors (63 cases of renal clear cell carcinoma, 31 cases of papillary renal cell carcinoma, 32 cases of chromophobe cell carcinoma, 58 cases of AMLmf, and 14 cases of oncocytoma), who underwent surgical resection in our hospital during the same period. Benign tumors accounted for 22.73% in our study, of which AMLmf accounted for approximately 64.44% of benign tumors, which is roughly the same as reported in the literature (20, 23).

The study had some limitations. First of all, the total sample size was not very large, mainly because of the low incidence of AMLmf, and we had only chosen patients who had surgery in our hospital without multicenter cooperation, which may increase the risk of overfitting. Working with more medical centers to obtain more cases will lead to better models. Second, we did not select all the clear cell carcinomas operated in our hospital at the same period, but only selected the cases with radiographic manifestations similar to those of AMLmf in this group of patients, which were not particularly typical clear cell carcinomas on CT images. Third, the cases included in this study had a large time span, and different CT machines and scanning technologies were used, which was consistent with the clinical practice. However, the early scanning equipment and technologies may have reduced the overall diagnostic accuracy. Third, the cases included in this study had a large time span, and different CT machines and scanning technologies were used, which was consistent with the clinical practice. However, the early scanning equipment and technologies may have reduced the overall diagnostic accuracy.

In conclusion, the machine learning model established by radiomics features extracted from multiphase MSCT images can be used to identify AMLmf from other renal masses. These models are expected to assist manual diagnosis and improve work efficiency and accuracy, which is a noninvasive and effective method for differential diagnosis.

## Figures and Tables

**Table 1:** The classification performance of the models using features from different phases.

Phase	AUC	Sensitivity	Specificity	Accuracy
PCP	0.7500	0.7143	0.7222	0.7167
CMP	0.8492	0.7619	0.7778	0.7667
NP	0.8254	0.7857	0.7778	0.7833
EP	0.7394	0.7381	0.7222	0.7333
ALL	**0.9193**	**0.8095**	**0.9444**	**0.8500**

## Data Availability

The patient data used to support the findings of this study are restricted by the Institutional Review Board of Xijing Hospital in order to protect patient privacy.

## References

[1] Mazziotti S., Cicero G., D’Angelo T. (2017). Imaging and management of incidental renal lesions. *BioMed Research International*.

[2] Jonasch E., Gao J., Rathmell W. K. (2014). Renal cell carcinoma. *BMJ*.

[3] Lee H., Hong H., Kim J., Jung D. C. (2018). Deep feature classification of angiomyolipoma without visible fat and renal cell carcinoma in abdominal contrast-enhanced CT images with texture image patches and hand-crafted feature concatenation. *Medical Physics*.

[4] Jinzaki M., Akita H., Oya M. (2017). Imaging features of renal cell carcinoma differential diagnosis, staging, and posttreatment evaluation. *Renal Cell Carcinoma*.

[5] Harisinghani M. G., Maher M. M., Gervais D. A. (2003). Incidence of malignancy in complex cystic renal masses (Bosniak category III): should imaging-guided biopsy precede surgery?. *AJR. American Journal of Roentgenology*.

[6] Wang R., Wolf J. S., Wood D. P., Higgins E. J., Hafez K. S. (2009). Accuracy of percutaneous core biopsy in management of small renal masses. *Urology*.

[7] Mues A. C., Landman J. (2010). Small renal masses: current concepts regarding the natural history and reflections on the American Urological Association guidelines. *Current Opinion in Urology*.

[8] Heuer R., Gill I. S., Guazzoni G. (2010). A critical analysis of the actual role of minimally invasive surgery and active surveillance for kidney cancer. *European Urology*.

[9] Zhang Y. Y., Luo S., Liu Y., Xu R. T. (2013). Angiomyolipoma with minimal fat: differentiation from papillary renal cell carcinoma by helical CT. *Clinical Radiology*.

[10] Kim M. H., Lee J., Cho G., Cho K. S., Kim J., Kim J. K. (2013). MDCT-based scoring system for differentiating angiomyolipoma with minimal fat from renal cell carcinoma. *Acta Radiologica*.

[11] Sung C. K., Kim S. H., Woo S. (2016). Angiomyolipoma with minimal fat: differentiation of morphological and enhancement features from renal cell carcinoma at CT imaging. *Acta Radiologica*.

[12] Ishigami K., Pakalniskis M. G., Leite L. V., Lee D. K., Holanda D. G., Rajput M. (2015). Characterization of renal cell carcinoma, oncocytoma, and lipid-poor angiomyolipoma by unenhanced, nephrographic, and delayed phase contrast- enhanced computed tomography. *Clinical Imaging*.

[13] Varghese B. A., Chen F., Hwang D. H. (2018). Differentiation of predominantly solid enhancing lipid-poor renal cell masses by use of contrast-enhanced CT: evaluating the role of texture in tumor sub-typing. *AJR. American Journal of Roentgenology*.

[14] Yu H. S., Scalera J., Khalid M. (2017). Texture analysis as a radiomic marker for differentiating renal tumors. *Abdom Radiol.*.

[15] Leng S., Takahashi N., Gomez Cardona D. (2017). Subjective and objective heterogeneity scores for differentiating small renal masses using contrast-enhanced CT. *Abdom Radiol.*.

[16] Yan L., Liu Z., Wang G. (2015). Angiomyolipoma with minimal fat: differentiation from clear cell renal cell carcinoma and papillary renal cell carcinoma by texture analysis on CT images. *Academic Radiology*.

[17] Zwanenburg A., Vallières M., Abdalah M. A. (2020). The image biomarker standardization initiative: standardized quantitative radiomics for high-throughput image-based phenotyping. *Radiology*.

[18] Huang Y., Liang C., He L. (2016). Development and validation of a radiomics nomogram for preoperative prediction of lymph node metastasis in colorectal cancer. *Journal of Clinical Oncology*.

[19] Ma Z., Fang M., Huang Y. (2017). CT-based radiomics signature for differentiating Borrmann type IV gastric cancer from primary gastric lymphoma. *European Journal of Radiology*.

[20] Yang C. W., Shen S. H., Chang Y. H. (2013). Are there useful CT features to differentiate renal cell carcinoma from lipid-poor renal angiomyolipoma?. *AJR. American Journal of Roentgenology*.

[21] Campbell S. C., Novick A. C., Belldegrun A. (2009). Guideline for management of the clinical T1 renal mass. *The Journal of Urology*.

[22] Fujii Y., Komai Y., Saito K. (2008). Incidence of benign pathologic lesions at partial nephrectomy for presumed RCC renal masses: Japanese dual-center experience with 176 consecutive patients. *Urology*.

[23] Snyder M. E., Bach A., Kattan M. W., Raj G. V., Reuter V. E., Russo P. (2006). Incidence of benign lesions for clinically localized renal masses smaller than 7 cm in radiological diameter: influence of sex. *The Journal of Urology*.

[24] Jeon H. G., Lee S. R., Kim K. H. (2010). Benign lesions after partial nephrectomy for presumed renal cell carcinoma in masses 4 cm or less: prevalence and predictors in Korean patients. *Urology*.

[25] Bosniak M. A., Megibow A. J., Hulnick D. H., Horii S., Raghavendra B. N. (1988). CT diagnosis of renal angiomyolipoma: the importance of detecting small amounts of fat. *AJR. American Journal of Roentgenology*.

[26] Chandarana H., Rosenkrantz A. B., Mussi T. C. (2012). Histogram analysis of whole-lesion enhancement in differentiating clear cell from papillary subtype of renal cell cancer. *Radiology*.

[27] Raman S. P., Chen Y., Schroeder J. L., Huang P., Fishman E. K. (2014). CT texture analysis of renal masses: pilot study using random forest classification for prediction of pathology. *Academic Radiology*.

[28] Feng Z., Rong P., Cao P. (2018). Machine learning-based quantitative texture analysis of CT images of small renal masses: differentiation of angiomyolipoma without visible fat from renal cell carcinoma. *European Radiology*.

[29] Lee H. S., Hong H., Jung D. C., Park S., Kim J. (2017). Differentiation of fat-poor angiomyolipoma from clear cell renal cell carcinoma in contrast-enhanced MDCT images using quantitative feature classification. *Medical Physics*.

[30] Fehr D., Veeraraghavan H., Wibmer A. (2015). Automatic classification of prostate cancer Gleason scores from multiparametric magnetic resonance images. *Proc Natl Acad Sci USA.*.

[31] Xu X., Liu Y., Zhang X. (2017). Preoperative prediction of muscular invasiveness of bladder cancer with radiomic features on conventional MRI and its high-order derivative maps. *Abdom Radiol.*.

[32] Cui E. M., Lin F., Li Q. (2019). Differentiation of renal angiomyolipoma without visible fat from renal cell carcinoma by machine learning based on whole-tumor computed tomography texture features. *Acta Radiologica*.

